# Cataphoresis vs. the Direct Application of the Galvanic Current for Obtunding Sensitive Dentine and How to Exclude Both

**Published:** 1898-06

**Authors:** W. G. A. Bonwill

**Affiliations:** Philadelphia, Pa.


### Cataphoresis vs. The Direct Application of the Galvanic Current for Obtunding Sensitive Dentine and How to Exclude Both.

BY W. G. A. BONWILL, D D S., PHILADELPHIA, PA.

Presented to the Section on Stomatology at the Forty-eighth Annual Meeting of the American Medical Association, held at Philadelphia, Pa., June 1-4, 1897.

When I speak of cataphoresis and the galvanic currents without medicaments, in 1856 to 1859, you will see that with all that has been done by the few workers in our profession, a quarter of a century had passed and no one thought there was anything in electricity until some one in medicine attempted to keep up with electricity in other branches and carry medicaments through the tissues as copper is supposed to be carried from one pole of a battery to the other as in electroplating; and now some dentist takes that up and tells us that cocain can be carried through the dense tissue of dentinal tubuli, and every one is ready to take it up as an insignia that makes it no better than an advertisement that he can perform all operations on the teeth painlessly. I remember, when I sent out a circular, on going into a new town in Delaware to get practice, what a commotion was set up and how every one was ready to try the new obtunder. Now after so many years have passed and this comes again to the light, do you wonder that I am more amused than chagrined?

At the meeting of the Odontological Society of New York, in October last, I presented before them the original patent gotten in 1859 for obtunding dentine and extracting pulps by the electric current alone. I made no effort at the time, nor since, to sell a single right to any one.

Now, what have I to say about cataphoresis? Is it a fact that a drug can be dissolved in water, or any other media, and be made to traverse the dentinal tubuli and enter the pulp chamber and produce an anesthetic or analgesic effect by osmosis? Or can it directly paralyze the sensitive dentine upon its surface and sufficiently deep to enable the operator to cut with impunity, painlessly, as is claimed for most obtunders now found in the market? All who support this, claim that some effect is produced through osmosis and electricity the power that enables the drug to produce its effect by direct circulation through the tubuli of dentine

I should not attempt to quibble over osmosis, or how or what does produce this supposed effect, but my long experience, dating further back than Dr. Richardson, of London, entitles me to ask what is the real agent in this so-called wonderful discovery after nearly one-half a century's burial.

There are so many means of influencing, not only the human being, but all animals below man, as to make them believe almost anything you wish, even to the complete annulling of pain, that we must be very conversant with past history in this line to enable us to say what agent has produced this so-called cataphoresis or, as with other pain annullers, what is the true philosophy or *modus operandi* in analgesia? It is a subject that has called forth the thought and research of men through all time, and there is still a mystery, though the agents are still appearing and the last is claimed as the best.

Osmosis, according to the best authorities, can only take place between two fluids of dissimilar natures or densities or gravities, where a porous membrane of tissue intervenes, or where a porous porcelain cup is used, as where salt of different specific gravity is used on either side of a porous membrane, when the fluids will in a short time, without any electricity, become of the same strength or gravity; or, in a Bunsen battery, where the porous porcelain cup that holds the bichromate solution, or nitric acid, and the glass or outer cup holding the sulphuric acid solution of 1 to 12, when the current is passed, what effect is produced on the liquids and what becomes of the bichromate or sulphuric acid? Do they pass around through the galvanic apparatus or coil and through the interrupter? You know this can not be. They are simply neutralized and lose their power to produce an electric current. How can copper be carried through a porous cup and be deposited upon the opposite pole? How can you take the fluid containing cocain and pass it through a membrane that is not porous? Dentine is porous only when the tooth has been extracted and dried and is void of all organic matter. So long as it is in the mouth it is full of fluid that is not interchangeable by osmosis, unless you can produce in the pulp chamber and canal a different density to the fluids in the peridentium. If equilibrium exist between this medium of dentine on either side of it, then there is a *statu quo* condition and no osmosis. You might as well tell me that a cup can be made of dentine to take the place of the ordinary porous porcelain or burnt clay cup in a Bunsen battery.

It is well to try to alleviate pain in any operation, but when all this can be done without it, and the patient is enabled to see that dentistry is not the inhuman thing dentists would have them conceive, and have them feel and know that they can be taught to bear all the pain consequent upon any operation upon the teeth, in excavating or removing pulp why should you not adopt means long ago in your grasp.

From the many experiments of others in electro-therapeutics, as far back as 1860 osmosis was proven to take place by a current of electricity through a porous membrane or diaphragm, but never through bone, either in the living or dead subject; and even when the osmosis was affected through a porous animal structure or membrane or porous cup, it was only done after many hours. Teeth were extracted by electricity, in this city, as early as 1859, perhaps 1856, but it was only by the shock produced at the instant the forceps were applied and which produced a diversion of the will force, by causing a sudden and violent inhalation into the lungs, and while the lungs remained inflated, the effect was good, for the senses were for the instant submerged or subjugated.

This was taken up quickly by the profession everywhere, but it failed because dentists generally had no idea of the agent or how to use it, or why it produced the effect. They did not see its simple philosophy. They supposed it was the potential and specific effect of the galvanic battery current with an interrupter. But this was not true in the manner in which it was practiced. Thousands of batteries were sold, and ignorance of the real cause of success made it a curse rather than a blessing.

I bought an instrument, such as was to be had then and which was recommended, and it worked well for a single extraction, but not when I had several teeth to remove, as I was compelled to do at that time in the country, for I had no experience nor the means to save human teeth. I soon found that the public preferred extraction because it was cheaper. But from my use of the battery and by becoming familiar with its working upon myself and patients, I found that the continuous current, or when interrupted several thousand times a minute, would annul pain when the positive pole was directly applied to the tooth in excavating and the negative pole on the face or in the hand with no dam or other agent.

All of this work, however, was preceded by experiments in the use of chloroform directly upon myself. That I may have you understand the steps leading up to the discoveries and principles involved, and the cause of failures in so many otherwise good agents that are overlooked and abandoned; and why I assert that all this cataphoresis in dentine is only the work of the current pure and simple; and how many agents we have had at our disposal that, had dentists only learned to apply scientifically, we would not be trying everything offered as obtunders or anesthetics or analgesics.

I had learned the use of chloroform and ether from my father, and particularly from an M.D. who was exploiting these new agents and also had a galvanic battery. At that time (1856) it was not known that chloroform had any other than an anesthetic effect. It was not discovered that it could be taken to a degree or in quantity to produce what is now known as analgesia.

You will now see the first dawning in my mind of the nature of these annihilators of pain, for the application was first made upon myself, and was administered by myself, which led to the discovery that chloroform could be taken to that extent that I could excavate my own carious cavities without pain and yet be sensible of the sense of touch and ability to perform the operation upon myself. It was an amazing thing to find that while the sensitive dentine was so obtunded that I could cut it with impunity, yet the special sense of hearing and touch was exaggerated. The excavator seemed to me as large as a hoe and the cavity as capacious as a bushel basket. There was no pain and my will sense was not subjugated, only the voluntary mind had been partially annulled. I could scarcely believe my own senses. Yet several separate trials convinced me that I had discovered a new property or rather phenomenon—the analgesic effects of ether and especially of chloroform.

Chloroform, while it would do what I wanted, would make my patients too sick and I had to abandon its use.

It was then that the battery, for extractions alone, was first brought out in Philadelphia. I mentioned above the philosophy of shock in its action on respiration, and soon had it exemplified to the satisfaction of the patient that, while electricity would annul pain in dentine, and living pulps (if not inflamed) could be removed, yet the too strong application of the current gave the patient such a severe shock while excavating that a violent inspiration led me to exclaim, "nature's anesthetic," and I then saw it was diversion of the will power, for when the lungs were being inflated so violently, the will could not take cognizance of actual pain. It was for the instant complete. Let any of you hurt a finger and how soon it is put in the mouth and a violent inhalation taken several times until pain is relieved. The infant in crying violently, while in pain from an accident, is relieved and falls to sleep from the constant sobbing and increased inspiration. All temporary teeth I extracted by this one sudden inhalation or diversion of the will, without a tear nor complaint. Two or three can be extracted while the breath is held in the lungs.

This led to the discovery that rapid breathing for sixty to ninety seconds, at the rate of 100 to the minute, would produce such an obtundity of the nerves and nerve centers that analgesia would be produced and the patient still be conscious of touch. This enabled me to perform all cases of excavating—extracting both teeth and the bony pulp, as well as all minor operations in surgery. A single respiration quickly taken and the breath held in the lungs will suffice for many trifling operations and all that is needed in extracting the temporary and permanent teeth of children. This was the birth of analgesia, in 1856, as proven upon my own person while operating upon myself.

This property of all anesthetics is very little known by medical men, and they have missed a very imporant part of anesthesia. Analgesia should be better understood and the application of rapid respiration would come into use in thousands of minor cases of operations where the custom is to completely anesthetize the subject. Let surgeons and dentists learn how to produce this effect and ether, chloroform and gas would no longer be used in three-fourths of the cases. In many cases of midwifery it is all that is desirable.

Let any one imagine that they are blowing a fire to make it blaze, taking full inhalations and forcibly ejecting or blowing, which can be done at least 100 times a minute, and while the patient is doing this the operator should talk to him, say no pain will be produced and urging him to breathe in this manner until sixty seconds have passed, when the patient can not feel any pain, as the voluntary system is subdued. The pneumogastric nerve now says, you can not go on as life would become extinct. For the next three minutes the respiration is only about six to the minute, showing the blood has been over-oxygenated. The patient has to be urged to breathe. When this is done previous to using ether or chloroform only one-half the quantity is used, the effect is more profound and there is not as much danger. Since this discovery of the rapid breathing method, in 1872, although analgesia was discovered by me in 1856, and was the stepping-stone thereto, I have used no other means of painless surgery in dentistry.

Cataphoresis is not a fact as applied to sensitive dentine and should have no place in dentistry.

You now know why I abandoned electricity for obtunding sensitive dentine and extracting; for this revelation of how nature relieves gave me the clue to a brighter step which dentists have been slow to recognize as a fact. Had they done so, then you would not today be looking for any other agent in most of the cases it is our lot to have.

